# Ameliorative effects of *Corchorus olitorius* leaf ethanolic extract on letrozole-induced polycystic ovary syndrome (PCOS) in rats 

**DOI:** 10.22038/ajp.2025.25627

**Published:** 2025

**Authors:** Udoh Ekaette Sunday, Essiet Grace Akanimo, Victor Udo Nna, Umoren Inyang Arit, Ugbem Theophilius Ipeh, Lapeh Pierre Takem, Umiom Edem

**Affiliations:** 1 *Department of Pharmacology, Faculty of Basic Medical Sciences, College of Medical Sciences, University of Calabar, P.M.B. 1115 Calabar, Cross River State, Nigeria*; 2 *Department of Histopathology, University of Calabar Teaching Hospital, Calabar, Cross River State, Nigeria*; 3 *Department of Physiology, Faculty of Basic Medical Sciences, College of Medical Sciences, University of Calabar, P.M.B. 1115 Calabar, Cross River State, Nigeria*; 4 *Department of Public Health, Middlesex University, London, United Kingdom*

**Keywords:** Hormonal imbalances, Hyperandrogenism, Exfoliative vaginal cytology, Polycystic ovarian syndrome (PCOS), Female fertility

## Abstract

**Objective:**

To evaluate the effects of *Corchorus olitorius* leaf extract on induced polycystic ovarian syndrome (PCOS) rats.

**Materials and Methods:**

36 female Wistar rats were divided into six groups (n=6) including Sham, PCOS + vehicle (Dimethyl sulfoxide), PCOS + Clomiphene citrate, PCOS + 200, 400, and 600 mg/kg *Corchorus olitorius*. The Sham group was administered CMC (carboxymethylcellulose) (0.5%) 1 ml/0.1 kg, while the PCOS groups were administered letrozole (1 mg/kg) dissolved in 0.5% CMC solution for 21 days via oral gavage. The PCOS condition was established to be successful when Papanicolaou-stained vaginal cytology of days 13-21 showed a greater dominance of clusters of empty cornified squamous cells. The duration of treatments was for 14 days ( via oral gavage) and euthanization occurred on the 15^th^ day. Hormonal levels, glucose level, lipid profile, hematological indices, liver function, kidney function, and histopathological studies of the ovaries and uteri were all determined.

**Results:**

PCOS induction led to abnormalities in hormonal levels, lipid profile, glucose levels, ovarian morphology, uterine morphology, and vaginal cytology of the PCOS rats compared to the Sham group (p<0.05). *C. olitorius *leaf extract showed its ameliorative effects in terms of normalizing the altered vaginal cytology, restoring most parameters, and improving the appearance of ovarian and uterine morphology.

**Conclusion:**

These results suggest that *C. olitorius* leaves ameliorate PCOS symptoms in the studied biochemical and histological parameters.

## Introduction

Polycystic ovarian syndrome (PCOS) is a complex endocrine and metabolic disorder of heterogeneous origin that affects about 9 – 18% of women in their childbearing ages (Kafali et al., 2004; Paixao et al., 2017). The term "polycystic ovarian syndrome" refers to the existence of small benign and painless cysts in the ovaries, which are medically expressed by a variety of symptoms and hormonal abnormalities, according to Paixao et al., (2017). These symptoms are classified clinically into acute and chronic effects based on the duration of their manifestations (Rosenfield and Ehrmann, 2016). The acute symptoms include excessive weight gain, insulin insensitivity, hyperandrogenism, hormonal imbalances, numerous polycystic ovaries, irregular uterine bleeding, anovulation, infertility, and many other identifying factors (Rosenfield and Ehrmann, 2016). Its chronic consequences include an increased risk of endometrial cancer, type 2 diabetes mellitus, dyslipidaemia, hypertension, ovarian torsions, and cardiovascular disorders (Franks, 2008). In addition, Petta et al. (2017) in a cohort of PCOS and non-PCOS women showed that the risk of liver steatosis and fibrosis was doubled in PCOS women whereas their renal function tests were within the normal limits.

Several explanations have been offered over the years about the pathologic causes of PCOS ranging from neuroendocrine, metabolic, and ovarian abnormalities in a self-perpetuating vicious cycle. Also, interactions between specific hormones, genes, and environmental stresses are among the commonly postulated pathways (Wilcox, 2005; Marcondes et al., 2015; Paixao et al., 2017). Gonadotrophic hormones such as luteinizing hormone (LH) and follicle-stimulating hormone (FSH), as well as prolactin, estrogen, progesterone, and testosterone aberrant secretions, have been reported to also play a role in PCOS pathogenesis (Wilcox, 2005; Falsetto et al., 2009; Paixao et al., 2017). PCOS women with genetic defects in insulin sensitivity also commonly have insulin resistance as the metabolic abnormality that leads to hyperinsulinemia (Wilcox, 2005; Falsetto et al., 2009; Jahromi et al., 2016). Hyperinsulinemia explains PCOS pathogenesis because insulinemia enhances steroidogenesis via its synergism with insulin-like growth factor-1 (IGF-1) in the pituitary gland. These result in LH secretions and a concomitant increase of LH actions on ovarian thecal cells to produce more androgens (Wilcox, 2005; Paixao et al., 2017). Consequently, the hyper-secreted androgens testosterone, androstenedione, and dehydroepiandrosterone (DHEA) lead to premature ovarian follicle atresia, multiple cysts formation, anovulation, and subsequent infertility state of PCOS. Furthermore, the persistent aromatization of androgenic hormones in obese conditions, increases estrogenic levels in the body, thereby further impairing the hypothalamic-pituitary gonadal (HPG) axis functions (Wilcox, 2005). HPG axis function dysregulation results in aberrant gonadotropin secretions with a consequent elevation and depletion of LH and FSH levels, respectively. Ultimately, PCOS women's LH to FSH level ratios are inverted from normal, with LH increasing, usually three times that of FSH (Wilcox, 2005; Shermin et al., 2019). 

PCOS is also acclaimed to be heritable, as reported in a recent finding involving early utero-exposure to androgens and epigenetic reprogramming of foetal reproductive tissues that activate the HPG axis pathway (Gainder and Sharma, 2019). Consequently, persistent abnormalities of ovarian steroidogenesis and follicular developments without appropriate therapeutic interventions significantly worsen PCOS conditions and make PCOS women more prone to endometrial hyperplasia and carcinomas (Wilcox, 2005; Shermin et al., 2019). 

The management of PCOS is usually symptomatic since the disease is a syndrome comprising numerous symptoms thereby, leading to the administration of numerous drugs such as spironolactone, glitazones, clomiphene, and metformin amongst many others, for its treatment (Jahromi et al., 2016). Nonetheless, these drugs have been reported to have side effects after prolonged usage ranging from arthritis, joint or muscle pains, and psychological disturbances (Falsetto et al., 2009; Jahromi et al., 2016). Moreover, most fertility drugs are ovarian stimulators and are frequently associated with ovarian cysts, ovarian cancers, and ovarian hyperstimulation syndrome (OHSS) (Falsetto et al., 2009). Ovarian hyperstimulation syndrome (OHSS) is an amplified response to excess hormone activation that commonly arises as a long-term consequence of fertility medications. This syndrome is quite painful and often causes the ovaries to swell further (Falsetto et al., 2009; Jahromi et al.., 2016). Hence, there is a need to explore more indigenous medicinal plant-based therapies in which *Corchorus olitorius* is a classical example of such a plant (Soladoye et al., 2014).

*C. olitorius* leaf has been reported to have anti-androgenic, antidiabetic, anti-inflammatory, and antioxidative effects amongst other numerous benefits (Soladoye et al., 2014; Orieke et al., 2019; Udoh et al., 2024). Therefore, *C. olitorius* leaf was evaluated in a PCOS model in this study, for the first time, to scientifically validate its folkloric claim in the management of PCOS.

## Materials and Methods

**Plants**

*C. olitorius* leaves were purchased from a local farmland in Calabar Metropolis, Cross River State, South-South, Nigeria, authenticated, and given herbarium voucher number Herb/Bot/UCC/077. These leaves were shade-dried for six weeks and pulverized into coarse powder. The leaf powder (1800 g) was extracted in 96 % ethanol in a Soxhlet apparatus for 72 hr and oven-dried at 30^o^C into a gel with a consistent weight. The recorded yield was 78.19 g of ethanol leaf extract of *C. olitorius *(CO), giving a percentage yield of 4.34 %.

**Animals**

Female albino Wistar rats aged 8–10 weeks old, with an initial body weight of 99–110 g were used in the present study. They were purchased from the Pharmacology Department Animal House and kept in standard polyvinyl cages (n=6), fed with standard rat chow, and allowed to acclimatize for two weeks at a temperature of 27 ± 5^o^C and a 12/12 hr light/dark cycle. All rats used for this research were handled based on the guidelines of the National Institute of Health (NIH), and the study was conducted after obtaining ethical approval from the University of Calabar, Faculty of Basic Medical Sciences Ethical Committee (Reg. no: 053PHA3119).

**Exfoliative vaginal cytology observation**

Throughout the research duration, the rats’ estrous cycle was monitored daily at 9:00 a.m. via exfoliative vaginal cytology (EVC) procedure using the method described by Kafali et al. (2004). This procedure entailed the use of 0.2 ml of normal saline drawn with a rubber pipette and inserted into the rat’s vagina at a depth of about 5–10 mm. The fluid was flushed 2-3 times by suctioning method with the pipette and after each lavage, a drop was smeared onto a slide and allowed to air dry for about 30 min. Thereafter, the air-dried microscope slide was immersed in 70 % ethanol, rinsed in distilled water, air-dried again, and stained with Papanicolaou multichromatic staining (PAP) solution. Large nucleated epithelial cells, cornified squamous epithelial cells, and polymorphonuclear leukocytes were the three cell types detected in the smears. The PCOS condition was established when PAP-stained vaginal cytology of days 13–21 showed a greater dominance of clusters of empty cornified squamous cells against the normal squamous epithelial cells seen in a regular of 4-5 estrous cycle days. This confirmed the successful establishment of an arrested estrus cycle seen in PCOS rats (Kafali et al., 2004; Mclean et al., 2012).

**Induction of PCOS and study design**

Thirty-six female albino Wistar rats with 4–5 days of regular estrus cycle were randomly selected and grouped into 6 groups (n=6 each). The groupings were Sham group, PCOS+ 2% Dimethyl sulfoxide (vehicle for *C. olitorius *extract), PCOS + clomiphene citrate (1 mg/kg)(positive control), PCOS + 200 mg/kg extract of *C.* *olitorus* leaf, PCOS + 400 mg/kg extract of *C. olitorus* leaf and PCOS + 600 mg/kg extract of *C. olitorus* leaf. The control group was administered with carboxymethylcellulose (CMC, 0.5 %) solution of a dose of 1 ml/0.1kg while the PCOS-induced groups were administered with letrozole (1 mg/kg) dissolved in 0.5 % CMC solution via oral gavage for 21 days (Kafali et al., 2004). The administration of treatments to the experimental rats lasted for 14 days via oral gavage.

**Food intake and body weight estimation in rats**

The feed intake in the different treatment groups was assessed daily all through the research duration. These feeds were weighed prior to their intake and their left-over were re-weighed and subtracted from their initial known quantity the next morning. In addition, the respective weight changes in the different experimental groups were noted weekly.

**Evaluation of blood glucose level**

A weekly glucose profiling was conducted using a glucometer (Finetest AC Premium Blood Glucose Monitoring System, Osang Healthcare, Korea) throughout the course of the research.

**Sample collection**

On the 35^th^ day of the study, the rats were fasted overnight, anesthetized via inhalation of chloroform vapor, and euthanized. Blood samples were collected by cardiac puncture for blood chemistry analysis. 

**Measurement of hematological and lipid parameters**

Hemoglobin (Hb) concentration, red blood cell (RBC) count, white blood cell (WBC) count, mean cell volume (MCV), mean corpuscular hemoglobin concentration (MCHC), and packed cell volume (PCV) were measured based on the comparative method of Ike et al. (2010).

Lipid profiling was performed for total cholesterol (TC), triglycerides (TG), high-density lipoprotein cholesterol (HDL-c), and low-density lipoprotein cholesterol (LDL-c) using the method of Vassualt et al. (1999).

**Evaluation of hormonal levels**

Estrogen, progesterone, testosterone, FSH, LH, and prolactin levels were estimated using CSB-E05110r, CSB-E07282r kit, CSB E05100r kit, CSB-E06869r, CSB E 12654r and CSB E06881r Cusabio kits (Wuhan Shi, China), respectively. The biochemical technique ELISA (competitive inhibition immunoassay) was used for this assay according to the experimental procedures of Fishchbach and Dunning (2015). 

**Determination of the liver and kidney function**

To assess liver function, serum levels of alanine aminotransferase (ALT), aspartate aminotransferase (AST), and alkaline phosphatase (ALP) were measured following the methods of Stevens (1966) modified by Gowda et al. (2009) and Avwioro et al. (2010). On the other hand, urea, creatinine, potassium, sodium, chloride, and bicarbonate were the kidney function parameters assayed in this study. These analyses were conducted using a ci8200 Integrated System (Abbott, USA) (Gounden and Jialal, 2011). 

**Histological studies**

The uterus and ovaries were fixed in 10 % formalin, trimmed into smaller portions, dehydrated in a graded series of ethanol, and cleared in xylene. Thereafter, they were embedded in paraffin wax, sectioned into paraffin sections of 3 µm, stained with hematoxylin and eosin (H & E) stains, and visualized under a light microscope at high power magnifications (×400). H & E-stained sections were analyzed using ImageJ software (ImageJ, NIH-Bethesda, MD, USA) for quantitative analysis.

**Statistical analysis**

Statistical analysis was conducted using one-way analysis of variance (ANOVA), followed by Tukey’s *post-hoc* test. GraphPad Prism software V8.0 (GraphPad, San Diego, CA, USA) was used for the analysis. The values of p<0.05 were considered significant. The results are expressed as mean±standard error of the mean (SEM).

## Results

**Effect of CO on estrus cycle of PCOS-induced rats**

At the onset of the study before PCOS induction, all rats had regular estrous cyclicity of proestrous, estrous, metetrous, and diestrous phases. For the Sham group group, all rats had regular estrus cyclicity all through the study. However, on days 13 – 21 of PCOS induction, 67, 67, 50, 50, and 67% of rats had irregular cyclicity of arrested estrus phases in the PCOS groups, respectively (Table 1). The PCOS + Clomiphene citrate and PCOS+600 mg/kg *C. olitorius* groups displayed improvement in irregular estrous cyclicity on days 25–35 (treatment weeks) relative to the PCOS + vehicle group.

**Table 1:** Effects of *C. olitorius* leaf extract on the percentage of rats/group with irregular estrus cycle in PCOS-induced rats

Duration	Sham group	PCOS + Vehicle	PCOS + CC (1 mg/kg)	PCOS + 200 mg/kg CO	PCOS + 400 mg/kg CO	PCOS + 600 mg/kg CO
Week 1	0	33.33	33.33	33.33	33.33	33.33
Week 2	0	50	50	50	33.33	50
Week 3	0	66.67	66.67	50	50	66.67
Week 4	0	83.33	33.33	50	50	33.33
Week 5	0	66.67	33.33	50	50	33.33

Values are percentages, n=6/group. CO: *Corchorus olitorius*, CC: clomiphene citrate, PCOS: polycystic ovarian syndrome.

**Effect of CO on reproductive characteristics of PCOS rats**

**Figure 1:**
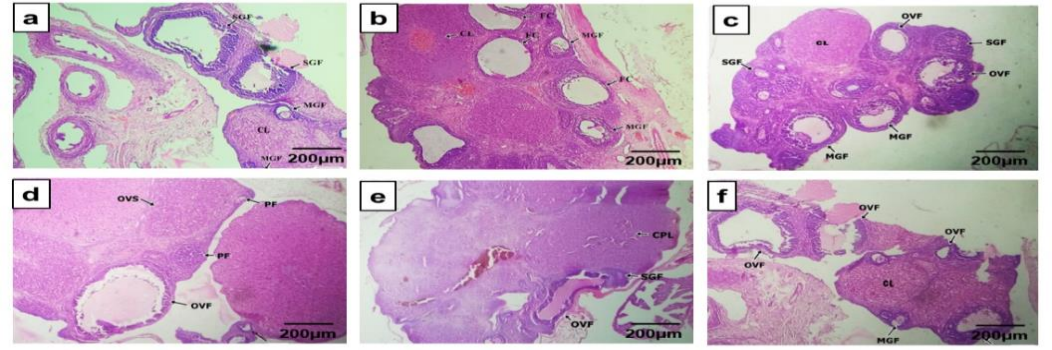
Transverse sections of rats’ ovaries in the different experimental groups. Staining was performed using the H & E technique, magnification ×400, scale bar: 200 µm. (a) Sham group, (b) PCOS + Vehicle (c) PCOS + Clomiphene citrate, (d) PCOS + 200 mg/kg CO*,* (e) PCOS + 400 mg/kg CO, (f) PCOS + 600 mg/kg CO*. *Section (a) has a normal outline of secondary Graafian follicle (SGF), mature Graafian follicle (MGF) and corpus luteum (CL). Sections (b) – (f) show the notable appearances of ovarian stroma/follicular cysts (FC) alongside SGF, MGF, and CL.

Ovarian weight increased (p<0.05) in the PCOS group relative to the Sham group. The PCOS-treated groups showed remissions (p<0.05) in ovarian weights at the end of the experiment respectively (Table 2). The effect of the different treatments on the rats also showed varying observations in the ovaries. In all the PCOS groups, the presence of ovarian cystic follicles (OVF/FC) was noted. The treated groups (Figure 1c-f) showed amelioration of the detrimental effects of PCOS induction after 14 days of treatment. In addition, the follicular cysts lumens in PCOS + vehicle had fewer layers of granulosa cells with thickened theca linings which were significantly reversed (p<0.05) in the PCOS + CC and PCOS + 600 mg/kg CO groups (Table 2). 

**Figure 2:**
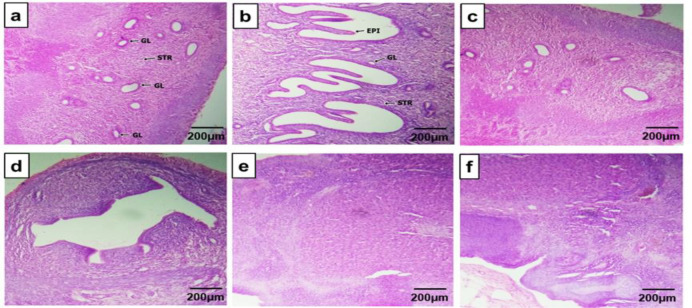
Transverse sections of rats’ uterus in the different experimental groups. Staining was performed using the H & E technique, magnification ×400, scale bar: 200 µm. (a) Sham group, (b) PCOS + Vehicle, (c) PCOS + Clomiphene citrate, (d) PCOS + 200 mg/kg CO, (e) PCOS + 400 mg/kg CO, (f) PCOS + 600 mg/kg CO. The Sham group shows the presence of numerous glands of varying patterns in the uterine stroma (a). The PCOS group shows glands of conglomerate formations with crypt-like appearance having more defined epithelium (b). Section (c) shows the defined epithelium of a blood vessel with the presence of glands. Section (d) shows the conglomerate of glands and diminished presence of uterine glands. Sections (e) and (f) show no conglomerate of glands and diminished uterine glands. EPI: epithelium, GL: gland, STR: stroma.

The uterine endometrium in the Sham group had a higher presence of numerous uterine glands (GL) compared to the PCOS groups (Figure 2a-f). The vehicle group (Figure 2b) showed glands of conglomerate formations with a crypt-like appearance, while the PCOS + CC, 400, and 600 mg/kg CO groups showed a reversal of these conglomerate formations of uterine glands (Figure 2c-f).

**Table 2:** Effects of *C. olitorius* leaf on reproductive characteristics of letrozole-induced PCOS rats

Parameters	Sham group	PCOS + Vehicle	PCOS + CC (1 mg/kg)	PCOS + 200 mg/kg CO	PCOS + 400 mg/kg CO	PCOS + 600 mg/kg CO
***Ovary morphometry***						
Relative ovaries weight (%)	0.46 ± 0.01	0.52 ± 0.01^a^	0.40 ± 0.01^a,b^	0.44 ± 0.01^b^	0.41 ± 0.00^b^	0.40 ± 0.01^a,b^
Ovary area (mm^2^)	37.10 ± 1.20	48.25 ± 1.34	40.64 ± 1.14	44.59 ± 1.19	45.96 ± 1.15	42.51 ± 1.56
CL number	3.94 ± 1.04	3.75 ± 0.81	4.86 ± 0.92	4.44 ± 0.97	4.02 ± 0.58	4.32 ± 0.84
GL thickness (µm)	80.99 ± 1.19	31.57 ± 1.41^a^	60.30 ± 0.75^a,b^	42.70 ± 0.99^a^	48.80 ± 1.16^a^	52.70 ± 0.78^a,b^
TL thickness (µm)	24.20 ± 0.73	51.75 ± 0.97^a^	32.47 ± 0.54^b^	50.30 ± 1.01^a^	49.90 ± 0.59^a^	41.40 ± 0.84
***Uterine morphometry***						
Myometrium area (mm^2^)	1.11 ± 1.12	4.77 ± 1.41^a^	2.88 ± 0.91	3.80 ± 0.90^a^	3.80 ± 1.29^a^	3.42 ± 1.62
Endometrium area (mm^2^)	2.53 ± 0.25	5.89 ± 1.99^a^	2.03 ± 0.28^b^	4.80 ± 0.44^c^	4.60 ± 0.66^c^	3.10 ± 0.29
Relative uterine weight (%)	0.61 ± 0.01	0.33 ± 0.01^a^	0.73 ± 0.01^b^	0.48 ± 0.01	0.51 ± 0.01^b,c^	0.63 ± 0.01^b,d^

Data are presented as Mean ± SEM, n = 6. ^a^ p<0.05 versus Sham group, ^b^ p<0.05 versus PCOS + Vehicle, ^c^ p<0.05 versus PCOS + CC, ^d^ p<0.05 versus PCOS + 200 mg/kg CO (Analyzed using one-way ANOVA followed by Tukey’s post-hoc test). CO: *Corchorus olitorius*, CL: corpus luteum, GL: granulosa layer, TL: theca layer.

**Effect of CO on hormonal levels of PCOS rats**

**Figure 3:**
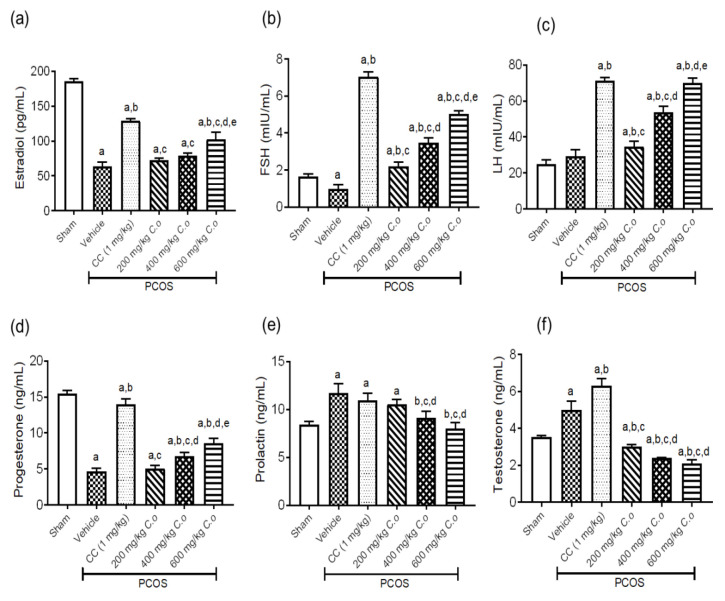
Effect of ethanolic leaf extract of *C. olitorius *on serum levels of (a) estradiol, (b) FSH, (c) progesterone, (d) LH, (e) prolactin, and (f) testosterone in PCOS rats. Values are mean±standard deviation, n = 6. ^a^ p<0.05 versus Sham group, ^b^ p<0.05 versus PCOS + Vehicle, ^c^ p<0.05 versus PCOS + CC, ^d^ p<0.05 versus PCOS + 200 mg/kg CO., ^e^ p<0.05 versus PCOS + 400 mg/kg CO. (Analyzed using one-way ANOVA followed by Tukey’s post-hoc test). CO:

The serum levels of estradiol, FSH, and progesterone decreased (p<0.05) in the vehicle group, but increased (p<0.05) in PCOS + CC and PCOS + CO groups compared to the normal group (Figure 3 a, b & d). Furthermore, the serum levels of LH, prolactin, and testosterone increased (p<0.05) in the PCOS groups. However, the levels of prolactin and testosterone decreased (p<0.05) whilst that of LH increased (p<0.05) in the 600 mg/kg CO group (Figure 3c, e and f). 

**Effects of CO on feeding habits and body weights of PCOS rats**

**Figure 4:**
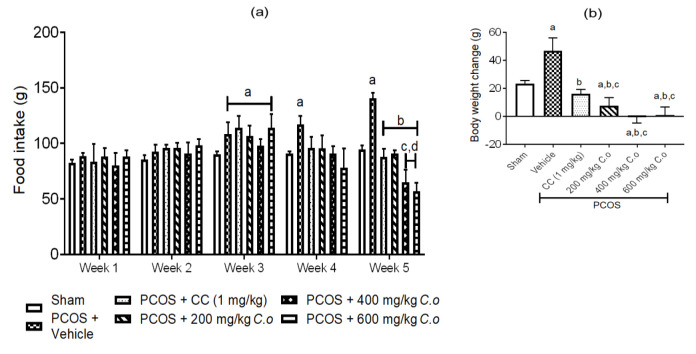
Effect of ethanolic leaf extract of *C. olitorius *on (a) food intake and (b) body weight change in PCOS rats. Values are mean ± standard deviation, n = 6. ^a^ p<0.05 versus Sham group, ^b^ p<0.05 versus PCOS + Vehicle, ^c^ p<0.05 versus PCOS + CC, ^d^ p<0.05 versus PCOS + 200 mg/kg CO*. *(Analyzed using one-way ANOVA followed by Tukey’s post-hoc test). CO: *Corchorus olitorius*, CC: clomiphene citrate.

On day 21 (Week 3), all PCOS groups had increased feeding habits, in relation to the Sham group. On day 35 (Week 5), all PCOS groups showed decreases (p<0.05) in feeding habits relative to the untreated PCOS group (Figure 4a). 

There was an increase in body weight change (p<0.05) in the untreated PCOS group compared to the Sham group (Figure 4b). However, all the treated PCOS groups showed decreased (p<0.05) body weight change relative to the PCOS untreated group, with the PCOS + CO groups lower (p<0.05) relative to the PCOS + CC group (Figure 4b).

**Effects of CO on blood glucose levels of PCOS rats**

The average initial blood glucose level at the onset of the study across all groups was 96.83 mg/dl. At the end of PCOS induction at week 3, there were significant increases in blood glucose levels, peaking as high as 139.68 mg/dl. These elevated blood glucose levels decreased steadily along the treatment weeks, and at week 5 in the 600 mg/kg CO group, there was a remarkable decrease (p*<*0.05) relative to other PCOS groups (Table 3).

**Effect of CO on biochemical indices of PCOS rats**

The serum lipid profile in the PCOS + vehicle group was elevated and statistically different (p<0.05) from all other treatment groups. PCOS + CC and CO-treated groups had significant decreases (p<0.05) in their lipid levels of TG, TC, VLDL, and LDL with the 600 mg/kg C. o group having a higher decline in the overall elevated serum lipid levels. HDL levels were significantly increased (p<0.05) in the CO-treated groups relative to the normal and PCOS + vehicle groups (Table 4).

AST, ALT, and ALP levels in the PCOS + vehicle group were all elevated (p<0.05) but decreased significantly (p<0.05) across all CO-treated groups in an increasing dosing manner (Table 4). Notably, treatment efficacies between the 600 mg/kg CO and PCOS + CC groups were statistically different (p<0.01) (Table 4).

Urea and creatinine levels of CO groups were elevated most especially the creatinine levels which had remarkably significant increases (p<0.01) at the 600 mg/kg CO.

 Na^+^ levels in the CO groups had no statistical significance (p>0.05) compared to both normal and PCOS + vehicle groups (Table 4). K^+^ and Cl^-^ levels of 600 mg/kg CO were statistically elevated (p<0.05) whereas HCO^3-^ level was significantly decreased (p<0.05).

**Effect of CO on hematological parameters of PCOS rats**

RBC, MCHC, MCV, and HB levels of the PCOS groups were statistically insignificantly different (p*>*0.05) from the Sham group (Table 5).

**Table 3:** Effects of *C. olitorius* leaf extract on blood glucose levels of letrozole-induced PCOS rats

Blood glucose level (mg/dl)	Sham group	PCOS + Vehicle	PCOS + CC (1 mg/kg)	PCOS + 200 mg/kg CO	PCOS + 400 mg/kg CO	PCOS + 600 mg/kg CO
Initial blood glucose	97.74 ± 0.22	95.58 ±0.61	94.86 ± 0.52	99.58 ± 0.39 ^a, b^	97.34 ± 0.76	95.9 ± 0.64 ^c^
Week 1	97.56 ± 0.47	107.46 ± 1.48	108.54 ± 0.34	103.39 ± 1.54	104.4 ± 1.14	104.58 ± 1.73
Week 2	101.52 ± 0.40	133.92 ± 3.51	133.38 ± 2.10	122.41 ± 3.84	108.54 ± 0.38 ^a ,b^	118.33 ± 2.73 ^b^
Week 3	90.18 ± 2.73	139.68 ± 1.69	135.1 ± 2.21	129.92 ± 4.66	126.51 ± 1.83 ^a^	126.4 ± 1.31 ^a^
Week 4	99.9 ± 0.66	123.48 ± 2.31	106.38 ± 4.93	107.86 ± 5.01	104.08 ± 2.74 ^a^	95 ± 2.47 ^a^
Week 5	99.36 ± 0.46	116.64 ± 3.14	89.82 ± 0.58 ^a^	83.84 ± 3.53 ^a^	78.44 ± 2.80 ^a^	61.74 ± 3.21^a, b, c^

Data are presented as Mean ± SEM, n = 6. ^a^ p<0.05 versus PCOS + Vehicle, ^b^ p<0.05 versus PCOS + CC, ^c^ p<0.05 versus PCOS + 200 mg/kg CO (Analyzed using one-way ANOVA followed by Tukey’s *post-hoc* test). CO: *Corchorus olitorius,* CC: Clomiphene citrate.

**Table 4:** Effects of *C. olitorius* leaf extract on biochemical indices of letrozole-induced PCOS rats

Parameters	Sham group	PCOS + Vehicle	PCOS + CC (1 mg/kg)	PCOS + 200 mg/kg CO	PCOS + 400 mg/kg CO	PCOS + 600 mg/kg CO
Lipid profile						
TC (mmol/L)	0.96 ± 0.03	1.91 ± 0.01	1.63 ± 0.02^a^	1.87 ± 0.01^,b^	1.49 ± 0.01^a,b^	1.09 ± 0.01^a,b^
TG (mmol/L)	0.39 ± 0.02	1.16 ± 0.06	1.06 ± 0.06^a^	1.19 ± 0.01^b^	1.05 ± 0.04^,a,c^	0.84 ± 0.07^a,b,c^
VLDL (mmol/L)	0.32 ± 0.02	0.59 ± 0.03	0.57 ± 0.03	0.66 ± 0.01^a,b^	0.49 ± 0.02^,a,b,c^	0.25 ± 0.02^,a,b,c^
HDL (mmol/L)	0.25 ± 0.02	0.43 ± 0.01	0.36 ± 0.01^a^	0.43 ± 0.01^b^	0.38 ± 0.01^a,c^	0.37 ± 0.02^a,c^
LDL (mmol/L)	0.37 ± 0.02	0.84 ± 0.01	0.68 ± 0.03 ^a^	0.90 ± 0.03^b^	0.61 ± 0.03 ^a,b,c^	0.49 ± 0.02 ^a,b,c^
Liver function						
AST (IU/L)	158.50 ± 4.04	214.25 ± 5.05	178.50 ± 6.6^a^	184.00 ± 3.80^a^	176.00 ± 3.80^ a^	55.30 ± 6.10^a,b,c^
ALT (IU/L)	80.20 ± 1.16	157.05 ± 2.58	93.50 ± 3.78	131.50 ± 2.4^a,b^	91.75 ± 2.90^a,c^	38.50 ± 2.70^a,b,c^
ALP (mmol/L)	170.51 ± 4.04	227.51 ± 4.44	190.01 ± 4.99^a^	214.02 ± 6.30	175.00 ± 6.30^a,c^	169.50 ± 6.90^a,b,c^
Kidney function						
Urea (mmol/L)	3.88 ± 0.32	4.55 ± 0.71	5.73 ± 0.42	4.63 ± 0.35	5.85 ± 0.25	6.61 ± 0.21
Creatinine (µmol/L)	75.80 ± 2.84	58.73 ± 3.5	65.40 ± 2.10	80.40 ± 1.50^a^	98.20 ±2.90^a,b^	108.20 ± 2.10^a,b,c^
Na^+^ (mmol/L)	138.30 ± 1.51	145.00 ± 1.01	149.70 ± 1.54	143.70 ± 1.54	140.30 ± 1.53	145.00 ± 0.58
K^+^ (mmol/L)	4.17 ± 0.15	4.67 ± 0.21	5.71 ± 0.11	5.87 ± 0.31	6.37 ± 0.21	7.23 ± 0.31^,a^
Cl^-^ (mmol/L)	103.30 ± 1.21	124.30 ± 1.53	114.70 ± 0.70	110.20 ± 1.16	112.10 ± 0.67	120.20 ± 1.16
HCO3^-^ (mmol/L)	28.67 ± 2.32	18.10 ± 0.57	14.67 ± 0.30	20.33 ± 0.57	17.10 ± 0.58	14.10 ± 0.54

Data are presented as mean ± SEM, n = 6. ^a^p<0.05 versus PCOS + Vehicle, ^b^p<0.05 versus PCOS + CC, ^c^p<0.05 versus PCOS + 200 mg/kg CO*. *(Analyzed using one-way ANOVA followed by Tukey’s *post-hoc* test). CO: *Corchorus olitorius*, TC: total cholesterol, TG: triglyceride, VLDL: very low-density lipoprotein, HDL: high-density lipoprotein, LDL: low-density lipoprotein, AST: aspartate transaminase, ALT: alanine transaminase, ALP: alkaline phosphatase, Na^+^: sodium ion, K^+^: potassium ion, Cl^-^: chloride ion, HCO_3_^-^: bicarbonate.

**Table 5:** Effects of *C. olitorius* leaf extract on hematological indices of letrozole-induced PCOS rats

Parameters	Sham group	PCOS + Vehicle	PCOS + CC (1 mg/kg)	PCOS + 200 mg/kg CO	PCOS + 400 mg/kg CO	PCOS + 600 mg/kg CO
Hb (g/L)	18.60 ± 0.03	18.80 ± 0.06	18.10 ± 2.89	18.50 ± 0.57	18.60 ± 0.03	18.30 ± 1.19
WBC (10^9^ cells/L)	11.40 ± 0.64	11.68 ± 2.22	12.81 ± 0.7	11.97 ± 0.77	14.16 ± 0.90	17.77 ± 0.3^a,b,d^
RBC (10^12^ cells/L)	7.32 ± 1.04	7.44 ± 0.32	7.23 ± 0.16	7.34 ± 1.11	7.36 ± 2.05	7.39 ± 0.90
MCHC (g/L)	34.30 ± 0.73	34.70 ± 0.66	35.40 ± 0.90	36.10 ± 1.09	36.40 ± 0.04	36.10 ± 0.63
MCV (f/L)	64.00 ± 0.79	66.00 ± 0.91	62.00 ± 0.01	63.00 ± 0.03	63.00 ± 0.03	64.10 ± 0.01

Data are presented as mean ± SEM, n = 6. ^a^ p<0.05 versus Sham group, ^b^ p<0.05 versus PCOS + Vehicle, ^d^ p<0.05 versus PCOS + 200 mg/kg CO. (Analysed using one-way ANOVA followed by Tukey’s *post-hoc* test). CO: *Corchorus olitorius*, Hb: hemoglobin, WBC: white blood cells, RBC: red blood cells, MCHC: mean corpuscular hemoglobin concentration, MCV: mean cell volume.

## Discussion

As earlier stated, PCOS is correlated with a myriad of symptoms ranging from distorted sex hormonal levels, inflamed ovaries, and metabolic disturbances amongst others. So far, no treatment has resolved the PCOS condition effectively; instead, they all aim to ameliorate the presented PCOS symptoms. In this study, we systematically studied the beneficial effects of* C. olitorius *leaf extract on biochemical and histological parameters of a PCOS state based on prior knowledge of *C. olitorius* effects. In a research study by Orieke et al. (2019), it was reported that *C. olitorius* leaf extract negatively affects male reproductive indices but leads to an excessive increase in FSH levels as also seen in this study. This decline in serum levels of testosterone and prolactin by CO was of great benefit to the PCOS rats in this study. 

CO also decreased the elevated relative weights of PCOS ovaries which have been attributed to its suppressive effects on ovarian steroidogenesis in animals (Gupta and Bhattacharya, 2003). However, it has a uterotrophic effect on PCOS uteri which was also observed by Reddy et al. (2016) and Rajan et al. (2017). In Figure 2 (Section D-F), CO uterotrophic effects led to an abundance of endometrial glands and these effects have been attributed to its ‘relaxant effects’ on uterine smooth muscles by Bafor et al. (2015) and Orieke et al. (2020), in which it was postulated to be effective against threatened miscarriages. Furthermore, numerous studies have shown the correlation between recurrent pregnancy losses and uterine endometrial gland deficiencies. This is because glandular secretions are necessary for uterine cell receptivity, decidualization, and eventual implantation of the blastocyst to its full-term growth in pregnancy (Baptiste et al., 2010; Orieke et al., 2020). CO extract (Figure 1 D-F) also improved the polycystic ovarian morphology (PCOM) although not in complete resemblance to the Sham group or PCOS + CC group. Nonetheless, the reappearance of healthy ovarian cortexes with more follicles having differentiated oocytes, granulosa cells, and theca cells is a positive indication. These curative effects of CO could be proposed to be a result of its metabolites’ antihyperglycemic, antioxidative, and antiandrogenic properties (Oboh et al., 2009; Orieke et al., 2019). Consequently, the re-emergence of squamous epithelial cells during daily vaginal smears at varying abundance at week 5 further showed the potency of the leaf (Table 1). 

In different relevant studies, *C. olitorius* leaf extracts have been found to have high contents of stearic acid and mucilage (Adon et al., 2018). Stearic acid has been reported in different studies to cause drastic weight loss in experimental animals due to its leptogenic effect (Adon et al., 2018). Leptin is a hormone produced by adipocytes and it helps in inhibiting hunger by upregulating the *STAT3* gene (Shen et al., 2014; Ujah et al., 2014; Jahromi et al., 2016). Furthermore, mucilage has been reported to inhibit pancreatic lipase activity, thereby leading to a decrease in visceral adipocytes in rats (Ameri et al., 2015; Ibrahim et al., 2016; Gainder and Sharma, 2019). These reported observations may be responsible for the observed body weight loss in CO-treated groups in the present study. In addition, *C. olitorius* leaf extract also demonstrated a hypoglycemic effect as demonstrated by lower fasting blood glucose levels in extract-treated groups, which was consistent with previous reports (Wang et al., 2011; Olusanya et al., 2018).

The AST and ALT enzymes are predominantly found in hepatocytes, RBCs, and kidneys, while ALP is specifically found in the hepato-biliary tract of the liver (Sachdeva et al., 2019). The levels of these enzymes are of clinical importance in ascertaining the integrity and functions of a viable liver. Elevations in ALT and AST ratios are synonymous with hepatocyte damage while elevations in ALP plasma levels are synonymous with hepato-biliary tract damage (Sachdeya et al., 2019). In the present study, 600 mg/kg CO decreased liver enzymes, which has been backed by some research by Omeje et al. (2016) and Ujah et al. (2014) on the hepatoprotective effect of C*. olitorius* leaf. This effect was attributed to its upregulation of hepatic antioxidative enzymes; δ‐aminolevulinic acid dehydratase (δ‐ALAD), catalase, and superoxide dismutase (Saliu et al., 2019).

PCOS may co-exist with other metabolic disorders, including dyslipidemia and CO has been reported to significantly lower TC, TG, and VLDL- and LDL-cholesterol whilst increasing HDL-cholesterol in an increasing dose manner (Omeje et al., 2016; Airaodion et al., 2019). Lipid profiling is an important clinical diagnostic tool for predicting cardiovascular disorders, in which a high LDL-c level is linked to a higher predisposition to atherosclerosis while a high HDL-c is linked to a reduced predisposition to atherosclerosis (Adon et al., 2018). We found that CO significantly decreased LDL-c level dose-dependently in PCOS rats and may be beneficial in decreasing atherogenic index in PCOS women with dyslipidemia.

Elevations in urea, creatinine, sodium, and chloride levels of *C. olitorius* leaf effects have been reported in numerous works by Mazumder et al. (2003), in which the elevated potassium levels were inferred from its abundance in the mineral composition of *C. olitorius* leaf. Whilst, increased chloride ion levels are usually in cohort with increased sodium levels and are synonymous with dehydration of the animal or possible renal impairment (Airaodion et al., 2019). Bicarbonate ion levels were decreased (p<0.05) in the PCOS groups and further diminished in CO-treated groups, which clinically signifies potential renal impairments that usually lead to metabolic acidosis. Similar work by Mazumder et al. (2003) has also reported on the potential side effects of high doses of *C. olitorius* leaves. In addition, these elevations could be attributed to the effect of elevated sex hormones on renal function parameters (Gupta and Bhattacharya, 2003; Cline, 2008). 

CO had no significant effects (p>0.05) on HB, RBC, MCHC, and MCV, thus indicating that there was no destruction of RBCs or stimulation of the erythropoietic processes while the significant increase (p<0.05) in the levels of WBCs suggests the possibility of immunological boosting effects of the extract (Adewusi and Afolayan, 2009). 

*C. olitorius* leaf’s myriad of bioactive phytochemicals can be hypothesized to be responsible for its ameliorative effects on the studied biochemical and histological parameters of a PCOS state, which is indicative of the great beneficial effects of *C. olitorius* leaf on female fertility.
